# Large Spatial Scale of the Phenotype-Environment Color Matching in Two Cryptic Species of African Desert Jerboas (Dipodidae: *Jaculus*)

**DOI:** 10.1371/journal.pone.0094342

**Published:** 2014-04-08

**Authors:** Zbyszek Boratyński, José Carlos Brito, João Carlos Campos, Maija Karala, Tapio Mappes

**Affiliations:** 1 Division of Ecology and Evolutionary Biology, Department of Biological and Environmental Science, University of Jyväskylä, Jyväskylä, Finland; 2 CIBIO/InBIO, Centro de Investigação em Biodiversidade e Recursos Genéticos, Universidade do Porto, Vairão, Portugal; 3 Departamento de Biologia, Faculdade de Ciências da Universidade do Porto, Porto, Portugal; BiK-F Biodiversity and Climate Research Center, Germany

## Abstract

We tested the camouflage hypothesis, or the linkage between animal (Saharan rodent) and habitat coloration, on the largest geographical scale yet conducted. We aimed to determine whether phenotypic variation is explained by micro-habitat variation and/or genetic polymorphism to determine 1) the strength of linkage between fur color and local substrate color, and 2) the divergence in fur coloration between two genetic clades, representing cryptic species, throughout the complete range of the African desert jerboas (*Jaculus jaculus*). We used a combination of museum and field-collected specimens, remote sensing tools, satellite and digital photography and molecular genetic and phylogenetic methods to investigate the above hypotheses. Along with showing that the two divergent genetic clades of jerboas occur sympatrically throughout their African distribution, we showed significant covariation between dorsal fur coloration of the animals and the color of their habitat. We also described significant phenotypic divergence in fur color, consistent with genetic divergence between the sympatric clades. The linkage between environment and phenotype supports the idea that the selection promoting cryptic coloration is persistent in contemporary populations of jerboas, however the phenotypic divergence indicates that it has different strengths (or optima) in the two clades. The mosaic distribution of micro-habitats occupied by geographically sympatric clades suggests that it may influence both ecological and evolutionary dynamics between these two cryptic species.

## Introduction

Spatial distribution and covariation between individual and environmental variability is a key issue in modern evolutionary ecology [1] [2]. Broad geographic distribution is expected to generate genetic patterns, if isolation by distance or geographic barriers is strong enough. With such isolation, differences can evolve between populations by drift alone, and can be mitigated by migration, gene flow and subsequent genome homogenization. However, in situations where local habitats form mosaics, geographic barriers are usually absent and the distance between populations is negligible. In these cases, divergence between populations evolves in response to local habitat variability, and diversification can be driven by ecological rather than spatial processes, even if the gene flow is present [3]–[5].

Coloration has been an important model for ecological and evolutionary studies since the early days of evolutionary theory [6] [7]. Darwin provided several examples of camouflage coloration, as evidence for adaptation, such as the color difference between black and red grouses [8]. Coloration quickly became a model system in evolutionary research [9]–[14] with the evolution of camouflage, or the covariation between the coloration of animals and their habitats, being one of the most striking examples of adaptation [15]–[20]. Theoretical studies of the evolution of camouflage coloration suggested that in areas of variably colored habitats, prey either reach a compromise between the two or more different micro-habitats or specialize in one of them, optimizing survival [10]. Compromise results in coloration that is intermediate between the micro-habitats, as in the desert lizard, *Sceloporus magister* [21] and a number of moth species [16], and may be the best strategy in some circumstances [22]. On the other hand, specialization results in color polymorphism, as in the mouse, *Peromyscus polionotus*, which consists of a dark-coated inland form and light-colored coastal populations [12] [23]. The trade-off between these two evolutionary pathways is shaped by resource availability and predation risk, features that often vary spatially across local micro-habitats and broad geographic areas.

The presence of coat color polymorphism in the model species studied here, the lesser Egyptian jerboa, *Jaculus jaculus*, has led to the description of a number of subspecies and even the proposition of separate species (i.e., *J. deserti*) [24]. It has been suggested that in *J. jaculus* dorsal fur color matches the background substrate and that animals living in gravel rock deserts are darker than populations inhabiting sandy desert areas and dunes [24]. This implies that camouflage adaptation to avoid predation has led to speciation, although substrate types and micro-habitats are assorted in the Sahara [25]. Since obvious topographic barriers that might promote population isolation and geographical genetic structure are missing within the African range of this species [26], migration is thought to homogenize adaptive responses.

The main aim of this study was to rigorously test on a large geographical scale if phenotypic variation, namely the polymorphism in dorsal fur color, can be predicted by variation in the color of the local micro-habitat, and if this phenotypic variation is linked to the geographic patterns of genetic polymorphism in lesser African jerboas [26]. We predicted that if natural selection has indeed favored camouflage in this species, we should detect (1) a significant correlation between dorsal fur and local substrate colors. Furthermore, if genetic clades (i.e., cryptic species) specialized to divergent micro-habitats and are still under selection promoting camouflage, then (2) a significant difference in fur coloration between them will be observed [27]. Whether these clades represent separate species or not, the ability to explore different, although assorted, micro-habitats would allow them to occur in sympatry over a large geographic scale. These different clades might also reflect variation in the level of the specialization to the micro-habitats, as indicated by strength of the correlation. To test the above predictions in a comprehensive way for these clades over such a large geographical scale, we developed a study based on satellite photography and remote sensing methods.

## Materials and Methods

### Specimens

**Figure 1:**
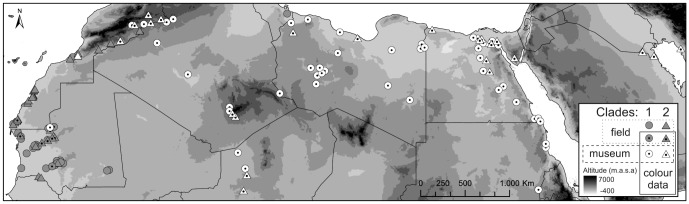
Sample locations of *Jaculus* specimens included in the study. Circles and triangles refer to clade 1 and 2, respectively. Grey and white figures refer to field and museum collected samples, respectively. Figures with dots refer to genetic samples accompanied by phenotypic data.

The lesser Egyptian jerboa (*Jaculus jaculus*) is mainly a nocturnal animal hunted by predators (birds of prey, owls) that use also visual clues for prey detection [15] [28] [29]. Samples of jerboas (85 specimens) were collected at the Smithsonian Natural History Museum, Washington, D.C., USA (69) and during field expeditions to Mauritania (16 road-killed individuals; see Appendix S1). Museum samples were originally collected as part of the African Mammal Project (1961–1972) and were preserved and stored without any chemical treatment as dried skins and skulls [30]. These samples represent the complete African distribution of the species (Fig. 1). Additional specimens (46) were included in phylogenetic analyzes (GenBank, *J. jaculus*: JN214504-JN214546, NC_005314, *J. orientalis*: JN652663, *Dipus sagitta*: AM407909 and *Allactaga elater*: AJ389534).

### Ethics statement

Ethical approval was not required for this study as all sampled animals originated from a museum collection or road-killed individuals. Museum sampling was conducted at the Smithsonian Institution, National Museum of Natural History, Division of Mammals, Washington, D.C., USA. Fieldwork was carried out with permission from the Ministére Délégué auprès du Premier Ministre Chargé de l′Environnement, Nouakchott, Mauritania (Permit: 460/MDE/PNBA). This permit was valid for all of Mauritania and no specific permissions were required for any of the sampled localities. Analyzes were done at the CITES registered laboratory: 13PT0065/S. IACUC approval was not requested as no animal was sacrificed and there were no animal husbandry, experimentation or care/welfare concerns.

### Molecular laboratory procedures

DNA from museum (tissues from inside skins and skulls) samples was extracted and purified using QIAamp (Qiagen) protocol. Polymorphic fragment of the cytochrome b gene, marker commonly used in phylogenetic and phylogeographic studies in mammals, was amplified and sequenced using primers designed for *Jaculus*: Jac1R (5′-TGCTGGTTTACAAGACCA-3′) and Jac4F (5′-CAAACCCACTTAATACGC-3′). The laboratory procedures were conducted in four stages with variable methods to account for the reduced quality of DNA in the museum tissues. DNA was amplified using the DreamTag kit. The 19 μl mixes contained 2 μl of DNA, 0.1 μl of DreamTag polymerase, 1.2 μl of each primer (1R and 4F), 0.4 μl of MgCl_2_ (3 mM), 2 μl of dNTP, 2 μl of reaction buffer and 10.1 μl of water. A PCR program with denaturation at 95°C, annealing (Ta) at 53°C and elongation at 72°C was maintained for 40 cycles. The results were confirmed with agarose gel electrophoresis. The PCR products were purified using the Exo-Sap procedure. Sequencing mixes contained 2 μl of purified PCR product, 1 μl of 3.2 μM primer, 0.5 μl of 5× BigDye sequencing buffer and 3.75 μl of 25× Ready Reaction Premix in 18 μl volume. Sequencing was conducted in Applied BioSystems 3130xl Genetic Analyzer. Unsuccessful samples were re-amplified with FailSafe. The 20 μl mixes contained 2 μl of DNA, 10 μl of FailSafe Premix F, 0.2 μl of FailSafe Entzyme Mix, 1.2 μl of 1R and 4F primers, and 5.4 μl of water. For amplification, a Touchdown PCR was used. Ta gradually lowered from 56 to 53°C during the first six PCRs to allow selective amplification. The following 31 cycles were kept at constant Ta. To ensure a sufficient concentration of DNA this step was repeated twice, with the end product of the first PCR used as the template for next one. The bands from agarose gels were cut, purified and sequenced with the Applied BioSystems analyzer as described above. The last 8 unsuccessful samples were amplified in 10 μl PCR mix (1 μ of DNA, 5 μl FailSafe Premix, 1 μl FailSafe Enzyme Mix, 0.6 μl of each primer and 2.7 μl of water) and a Touchdown PCR (56–52 °C) during the first six cycles with a total of 41 cycles. The PCR reaction was repeated using the PCR product as template. Sixteen field-collected samples were analyzed following published protocols [31].

### Phylogenetic analyzes

**Figure 2:**
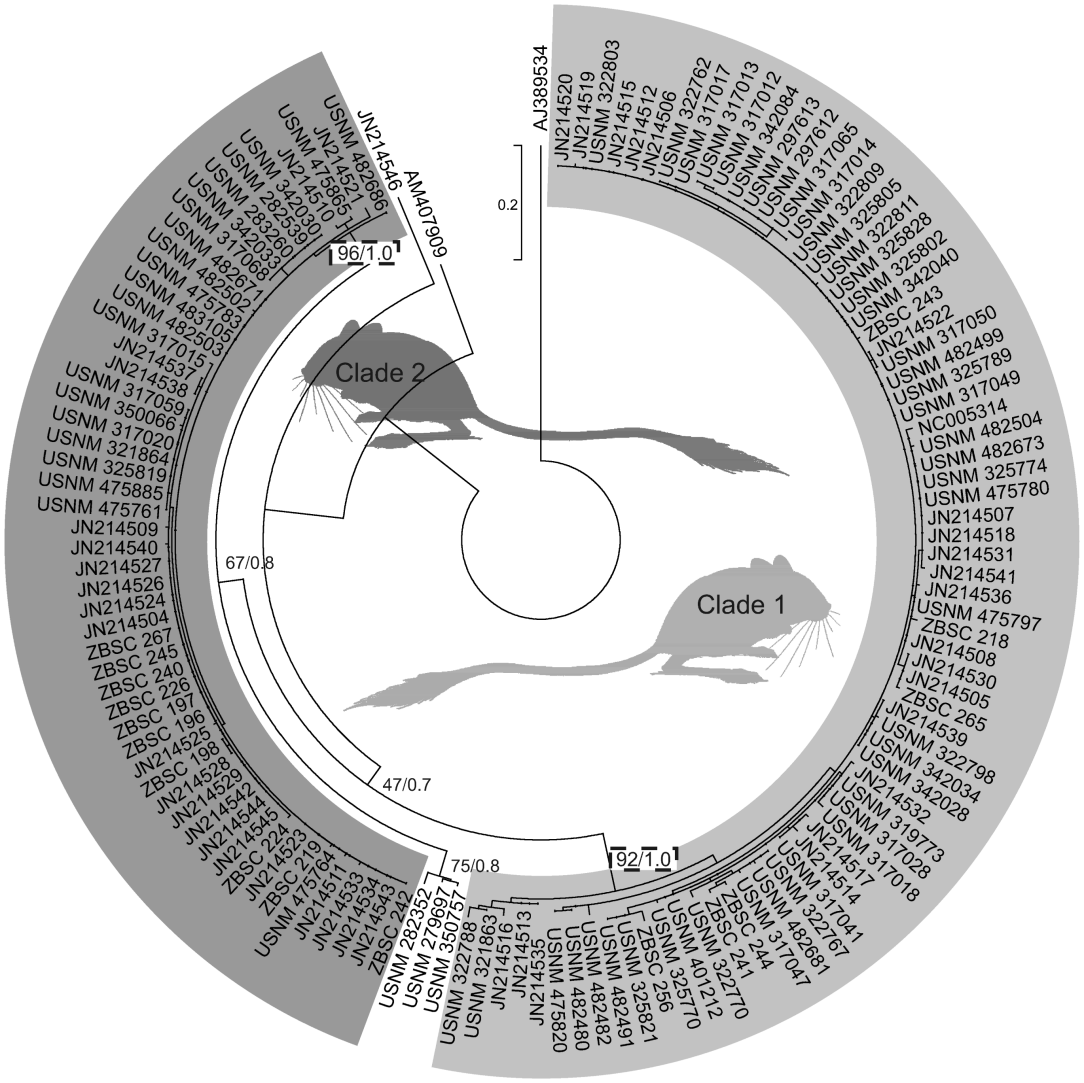
Maximum likelihood (ML) tree of a portion of the cytochrome b gene. Numbers in the external nodes refer to codes of the samples (see Appendix S1). Numbers in the internal nodes represent the percentage of bootstrap support for ML and posterior probabilities for Bayesian methods. Framed values represent statistics for the two main clades. The intensity of grey color of shadings and drafted animals is based on the overall dorsal fur luminosity of individuals pictured on the appended photograph (see Appendix S2).

The sequences were aligned and analyzed using SeqScape (Applied BioSystems) and ClustalX (www.clustal.org). Phylogeny was conducted with the Maximum Likelihood method (ML in PhyML 3.00 [32]), with the K80 model and the proportion of invariable sites (0.644) was selected with jModelTest 2 [33], and with the Bayesian method (BI) using GTR model with prior uniform distributions for the proportion of invariable sites (0.00, 1.00) and gamma shape parameter (0.00, 200.00; MrBayes 3.1.2; Fig. 2) [34]. Four Markov Chain Monte Carlo simulations were run for 10 million generations, starting from a random tree. The trees were sampled every 100^th^ generation with the first 30% of the trees discarded as burn-in. The remaining trees were used to construct a 50% majority consensus tree. The robustness of tree nodes was assessed by 1000 bootstrap replicates (ML) and posterior probabilities (BI). Descriptive statistics, tests of selective neutrality and mismatch haplotype distributions were calculated with Arlequin 3.11 (http://cmpg.unibe.ch/software/arlequin3).

### Animal coloration

Digital photography analysis was used to estimate animal coloration [35] only on specimens in suitable conditions (i.e., clean, without excessive dust). Museum (68) and field-collected (9) specimens were photographed alongside a ColorChecker (X-Rite, Michigan, USA) with a Canon EOS 350 digital camera. The high-resolution photographs (3888×2592 pixels) were saved in TIFF format and white balance was corrected using GIMP 2.0 (www.gimp.org) with the white reference of the ColorChecker. Fujifilm Hyper-Utility 2 (www.fujifilm.com) was employed to quantify coloration from a square-shaped area on each animal's uniformly colored back twice, independently. The squares, selected independently, and randomly, for two measurement trials, were as large as possible (2 cm^2^) while excluding the darker fur near the tail and the lighter fur occasionally showing from the sides or undersides of the animals. Luminosity and reflectance of red, blue and green were measured as a Red-Green-Blue (RGB, 8-bit) standard values (from 0 to 255). The museum archives provided geographic coordinates or descriptions of the collection location of the museum samples [30]. Field samples were georeferenced using the global positioning system (GPS).

### Environmental coloration

Remote sensing (RS) techniques were applied to estimate environmental coloration of sample locations from available NASA Landsat satellite images (landsat.gsfc.nasa.gov). A total of 90 satellite images from the Landsat 5 Thematic Mapper (TM) and Landsat 7 Enhanced Thematic Mapper (ETM^+^) were obtained through the Global Visualization Viewer (GLOVIS; glovis.usgs.gov; see Appendix S1). The time period of the 90 images (with spatial resolution of 30 m) corresponded to the dry seasons of 2003. To quantify repeatability we also used 90 images from 1999–2002 (see Appendix S1). Only images with cloud cover lower than 10% were analyzed. To eliminate atmospheric effects and obtain the real surface reflectance, an absolute atmospheric correction was performed through two steps: 1) conversion of 8-bit satellite-quantized calibrated digital numbers to at-satellite radiance; and 2) conversion of at-satellite radiance to atmospherically-corrected surface reflectance [36]. This atmospheric correction accounts for variations in Earth-Sun distance, sun angle, light diffuse irradiance, atmospheric transmittances and biases associated to the measurements of each satellite sensor (see detailed information in Supporting Information). Luminosity was calculated as a RGB weighted average using ITU-R BT.601 standard coefficients (International Telecommunications Union 2011: www.itu.int/rec/R-REC-BT.601-7-201103-I/en). The landscape reflectance was measured as average pixel value from a 10 kilometer radius around the sample origin. This accounted for species movement abilities (around 10 km per night) and for possible error emerging from inaccurate coordinates of pre-GPS samples. The radii of samples collected prior to local intensive infrastructure development were moved slightly to prevent man-made, irrigated areas or open waters from being included. All RS analyzes were developed in ArcGIS 9.3 [37]. The most intensive predation on jerboas is expected during evenings/mornings. Satellite images were taken in daylight and animal skins were photographed in full light conditions. Full light photography thus gives a relative, rather than direct, measurement of animal-habitat match [15] [19] [20], even if much efforet has been put to normalize variations in color attributes between satellite images and direct digital images of the animals.

### Statistical analyzes

Repeatability of fur and habitat coloration for the two independent measurements was estimated with the intraclass correlation coefficient (ICC) based on between and within group's mean squares from ANOVA [38]. Correlations between fur and environmental colorations were tested with partial Pearson correlation that included the origin of samples (either museum or field-collected). Variables potentially affecting animal coloration, including coloration of habitat, were tested with the mixed model procedure. The model importance and statistical significance of the effects were estimated with mixed model and Akaike information criterion (AIC) with *nlme* and *MuMIn* packages in R 3.0.1 (R Development Core Team, 2011). Fur luminosity and reflectance of red, blue and green were tested in independent analyzes as response variables, although colors have, with some exceptions [39], less biological meaning for nocturnal predators than overall luminosity. Individual ID was included in models as a random predictor, clade affiliation (with two levels of two genetic clades) as a fixed predictor, environmental luminosity and environmental reflectance of red, blue and green as continuous predictors, and factorial interaction between fixed effects and continuous predictors were included to construct the most complicated starting models. Fixed effects of continental affiliation (Africa versus Middle East), environment modification (unmodified versus urban, agricultural or near water, as detected from satellite images), sampling type (museum versus field) and their interactions with continuous predictors were also tested.

## Results

### Genetic variation

A total of 131 sequences (at least 352 base pairs long) covering the entire species range in North Africa were analyzed. Bar-codding ML and BI methods were consistent in showing two well-supported monophyletic clades (Fig. 2) with the broad distribution overlap (Fig. 1). The mismatch haplotype distributions for the African clades were closer to unimodality (Appendix S1), consistent with the Sudden Expansion Model (p > 0.4). Tajima's D and Fu's F neutrality tests were negative, with the exception of Tajima's D for the combined data set (Table 1). Estimated diversity indices (Table 1) and demographic parameters were slightly higher for African clade 1 [τ(CI)  =  3.4(0.7–12.5); Θ_0_(CI)  = 1.7(0–8.1); Θ_1_(CI)  =  24.7(6.9–∞)] than for clade 2 [τ (CI)  =  1.4(0–5.9); Θ_0_(CI)  =  1.0(0–5.9); Θ_1_(CI)  =  ∞(3.7–∞); based on 1000 replicates] but their confidence intervals overlapped. The clades differed by 16 fixed mutations, with an average number of nucleotide substitutions per site of 0.10.

**Table 1:** Sequence diversity and neutrality tests for cytochrome b sequences of lesser Egyptian jerboas. n_i_  =  number of samples, n_h_  =  number of haplotypes, n_p_  =  number of polymorphic sites, H  =  haplotype diversity, π%  =  nucleotide diversity.

	n_i_	n_h_	n_p_	H	π (%)	Tajima's D	p	Fu's F_s_	p
all	127	64	78	0.97	6.09(2.99)	1.15	0.91	−13.95	0.015
Clade 1	72	40	42	0.96	1.31(0.72)	−1.55	0.04	−25.70	<.0001
Clade 2	52	21	22	0.90	0.70(0.41)	−1.67	0.02	−14.27	<.0001
Clade 2_including Middle East_	55	24	38	0.91	1.26(0.70)	−1.62	0.03	−10.36	0.002

### Repeatability and correlations

**Figure 3:**
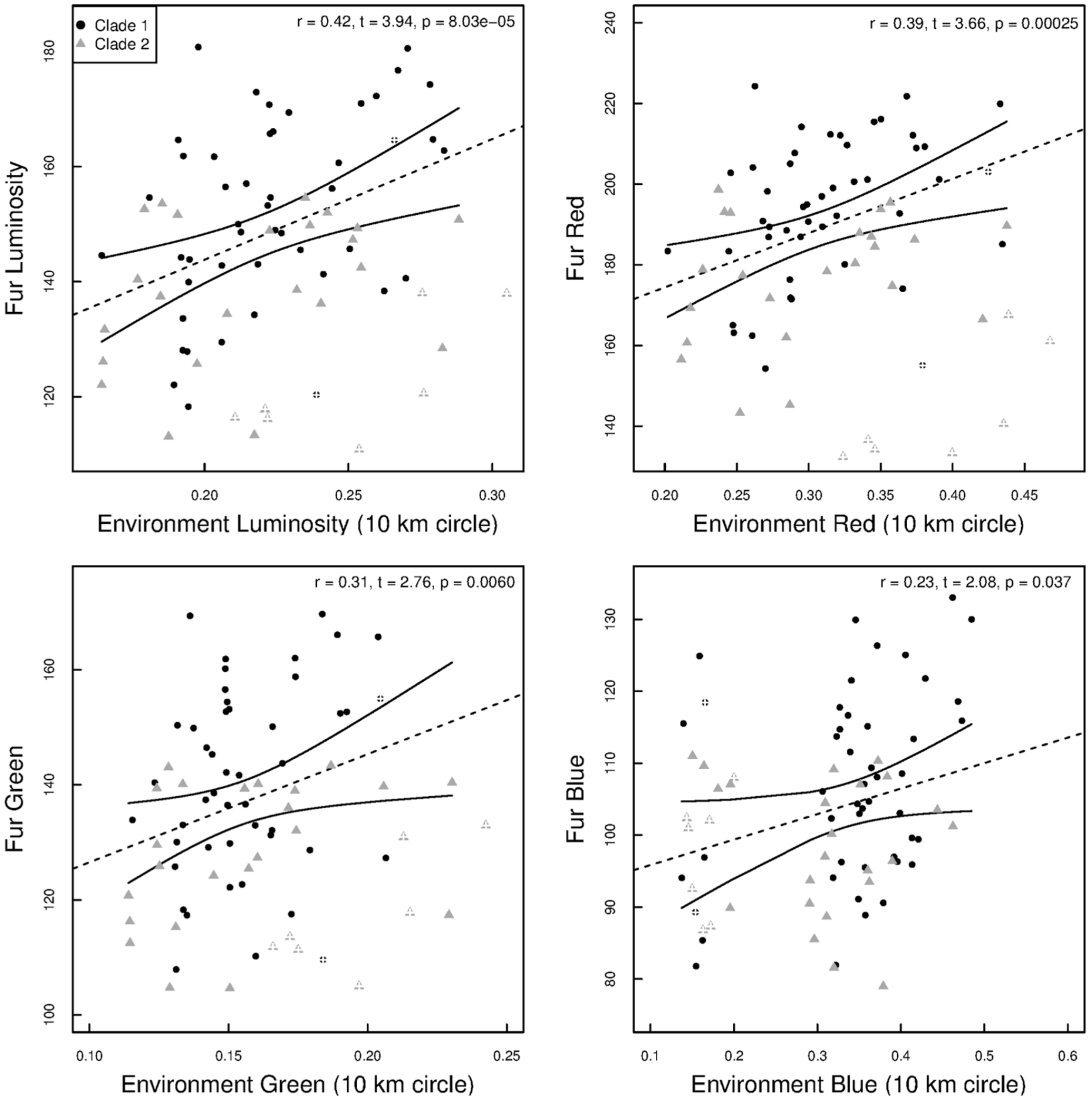
The association between individuals' dorsal fur and environmental luminosity. Circles (black) and triangles (grey) refer to clade 1 and 2, respectively. Figures with crosses refer to field collected samples. Statistics are based on partial Pearson correlations accounting for sample type, i.e., museum or field specimens.

The estimated luminosity and RGB colors of dorsal fur were highly and significantly repeatable, with an ICC of 0.98 (p < 0.001; separate RGB colors: 0.99, 0.98, 0.97; p < 0.001). ICC for luminosity of the environment was 0.75 (p < 0.001; separate RGB colors: 0.65, 0.68, 0.82; p < 0.001). Dorsal fur luminosity was significantly correlated with environmental luminosity, both for the complete dataset (Fig. 3) and for datasets including only animals belonging to clade 1 (partial correlations: r  =  0.45, t  =  3.36, df  =  47, p  =  0.0008) or clade 2 (r  =  0.40, t  =  2.24, df  =  30, p  =  0.025). Dorsal fur red, green and blue color bands were also significantly correlated (r ≥ 0.23, p ≤ 0.037; except for the blue band within clade 2 (p > 0.4) with environmental red, green and blue colors bands (see Appendix S1).

### Phenotypic and environmental variation and covariation

The mixed models showed consistent results among analyzes and highlighted the importance of clade affiliation and environmental coloration (RGB colors and luminosity) in predicting variability in the animals' dorsal fur luminosity (Table 2; see also: see Tables S3–S5 in Appendix S1 for consistent results of reduced datasets). The models explaining variability in dorsal fur coloration were consistent and always included the clade affiliation of the specimen and environmental coloration. The estimated relative importance of traits showed that clade affiliation and red and green bands of environmental coloration, as well as the type of the sample (either field collected or museum), were the traits strongest related to luminosity of dorsal fur (Table S5 in Appendix S1). As the differences between models were not strong (DELTA < 2; likely caused by correlations between color predictors) here we focused on the simplest, best models to satisfy parsimony. Analyzes with these mixed models showed red coloration of the environment and animals clade affiliation as the most consistent and significant predictors among different variables and analyzes (Table 3; Table S6 in Appendix S1). When the best models were analyzed within the clades separately, the significant effects of environmental coloration and the effect of sample origin (field versus museum) were maintained (for clade 1: red: t  =  3.37, p  =  0.0016, origin: t  =  −2.09, p  =  0.042; for clade 2: red: t  =  2.30, p  =  0.0297, origin: t  =  −4.02, p  =  0.0004).

**Table 2:** Model components and parameters from the AIC model selection procedure.

	Models parameters				
Dependent and independent variables	df	logLik	AIC	Delta	Weight
Fur Luminosity					
Clade+M/F+Red+Green	7	−290.5	628.8	0.00	0.29
Clade+M/F+Red+Blue+Luminosity	8	−285.3	629.8	1.00	0.18
Clade+M/F+Red+Blue+Green	8	−285.9	629.8	1.00	0.18
Clade+M/F+Red+Green+Luminosity	8	−283.7	629.8	1.00	0.18
Clade+M/F+Blue+Green+Luminosity	8	−284.7	629.8	1.00	0.18
Fur Red					
Clade+M/F+Red+Green	7	−299.4	647.8	0.00	0.27
Clade+M/F+Red+Green+M/F*R.	8	−292.7	648.0	0.23	0.24
Clade+M/F+Red+Green+M/F*G.	8	−292.2	648.4	0.64	0.2
Clade+M/F+Red+Green+C.*R.+C.*G.+M/F*R.	10	−278.9	649.1	1.28	0.14
Clade+M/F+Red+Green+C.*R.+M/F*R.	9	−287.0	649.2	1.37	0.14
Fur Blue					
Clade+Green+Luminosity	6	−277.9	595.2	0.00	0.29
Clade+Green+Luminosity+C.*G.+C.*L.	8	−264.2	595.8	0.55	0.22
Clade+Green+Luminosity+C.*L.	7	−271.9	596.1	0.94	0.18
Clade+Red+Blue	6	−281.1	596.2	0.94	0.18
Clade+Red+Blue+Luminosity	7	−273.9	596.8	1.53	0.13
Fur Green					
Clade+M/F+Red+Green	7	−290.2	628.3	0.00	0.23
Clade+M/F+Red+Blue	7	−292.9	628.5	0.19	0.21
Clade+M/F+Green+Luminosity	7	−289.9	628.5	0.24	0.21
Clade+M/F+Red+Blue+Luminosity	8	−284.9	628.9	0.58	0.17
Clade+M/F+Red+Blue+Green	8	−285.4	628.9	0.58	0.17

Fur luminosity and RGB colors, the dependent variables, were analyzed in separate mixed models that included clade (two clades) and museum/field (M/F) affiliations as fixed factors, luminosity (Luminosity) and RGB environmental coloration as continuous predictors with factorial interactions between them, and a random factor of individual code.

**Table 3:** Results for best models, for all significant effects, of mixed model analyzes for variation in dorsal fur luminosity and RGB colors.

Dependent	and independent var.	Value	SE	df	t	p
Fur Luminosity						
	Intercept	131.0	10.27	73	12.8	<.0001
	Clade	−13.1	3.35	73	−3.91	0.0002
	Field/Museum	−25.4	5.72	73	−4.44	<.0001
	Red	113.0	28.6	73	3.95	0.0002
Fur Red						
	Intercept	181.5	11.8	72	15.4	<.0001
	Clade	−16.8	3.8	72	−4.45	<.0001
	Field/Museum	−39.5	6.35	72	−6.21	<.0001
	Green	−396.1	184.4	72	−2.15	0.035
	Red	296.4	86.8	72	3.42	0.0010
Fur Blue						
	Intercept	80.0	9.6	73	8.29	<.0001
	Clade	−6.5	2.9	73	−2.44	0.017
	Green	−266.1	113.4	73	−2.35	0.022
	Luminosity	334.5	97.4	73	3.44	0.0010
Fur Green						
	Intercept	117.4	10.2	73	11.5	<.0001
	Clade	−11.6	3.3	73	−3.50	0.0008
	Field/Museum	−20.9	5.7	73	−3.68	0.0004
	Red	113.9	28.4	73	4.01	0.0001

Best models were depicted from the AIC model selection procedure.

## Discussion

It has been shown in previous studies on rodents that adaptive dorsal fur coloration can evolve rapidly [40] in response to selection pressures caused by predation [19] and that the molecular and structural mechanisms for coloration seem to be relatively labile [20] [41] [42] [43]. Though there are studies on geography of fur color polymorphisms, they have focused mainly on few local populations. Here, we developed a novel remote sensing methodology (based on publicly available resources and therefore easily applicable in other systems) to study camouflage over a large geographic scale (roughly the size of the Australian continent) that covered the complete African/Saharan range of our model species, the African desert jerboa (Figs 1 and 2) [26]. Previous research based on both mitochondrial and nuclear sequences showed no signs of introgression implying reproductive isolation between the two sympatric jerboa mitochondrial clades [26] [31]. These previous studies hypothesized, on one hand, that the clades coexist in geographical sympatry across the entire Sahara, but also that they segregate to distinct micro-habitats [24]. The implied adaptive mechanism behind the origin of these putative species is the evolution of camouflage, as a response to avoid avian predators [28] [29]. Both the differences in fur luminosity between the clades and the animal-habitat color match detected here support this mechanism (Tables 2 and 3). Alternatively color polymorphism between clades could be related to thermoregulation, as the differences of mean annual temperature between the two clades were documented [31], although it seems to be less likely as jerboas regulate temperature internally [44].

**Figure 4:**
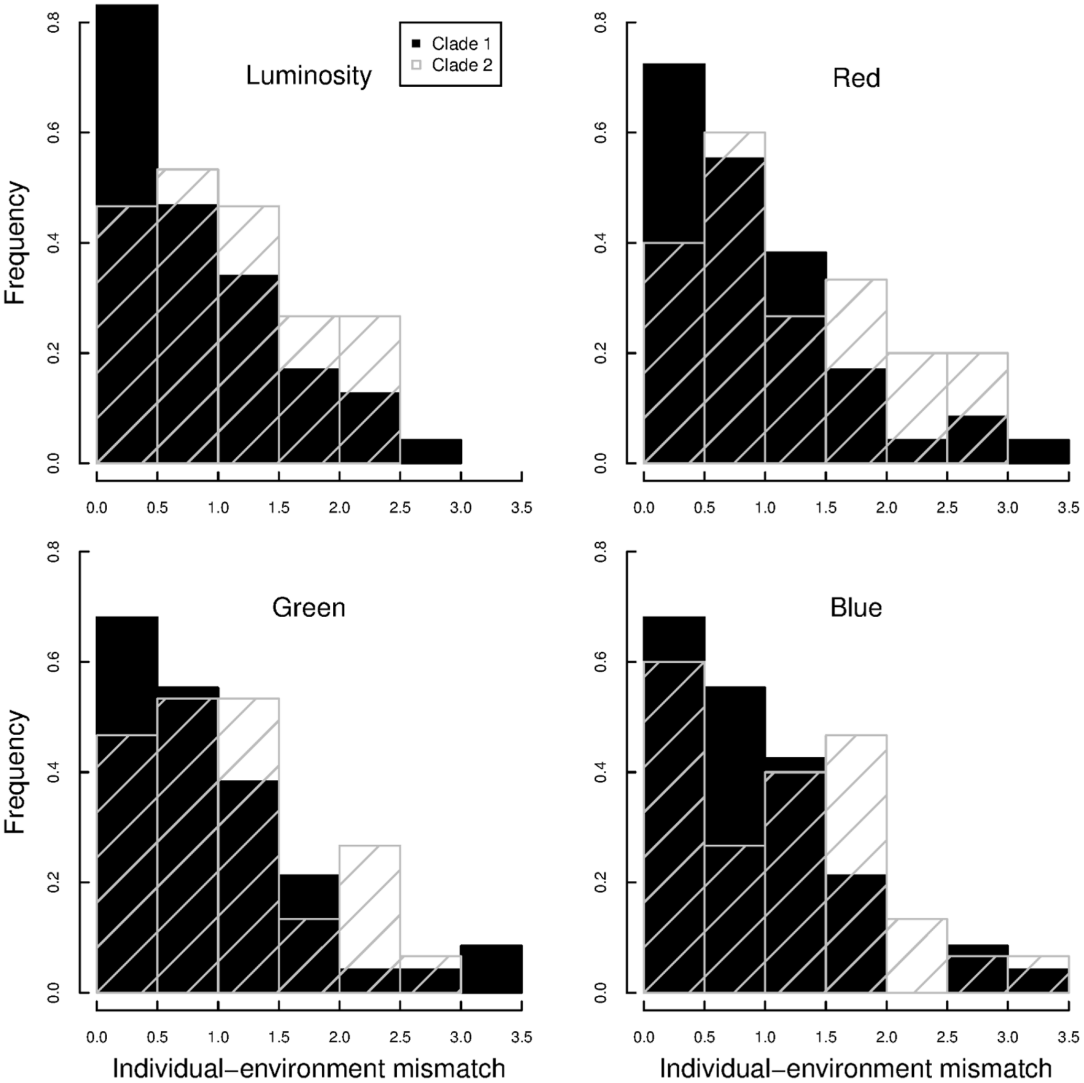
Distributions of mismatches between relative dorsal fur and environmental colorations. Black (filled) and grey (with diagonal lines) columns refer to clade 1 and 2, respectively.

The detected linkage between consistent (repeatable) environmental and phenotypic variability suggests that natural selection may be promoting cryptic coloration of dorsal fur and that this mechanism may have generated phenotypic divergence between the clades (Fig. 3). The observed correlation (r  =  0.42) implies that relatively strong selection pressures are shaping this phenotypic polymorphism (Tables 2 and 3), a result consistent with previous experimental studies on other rodents [15] [19]. Although the statistical interactions between clade and environmental effects were not significant (Table 2), the correlation between phenotypic and environmental luminosity in clade 1 (r  =  0.45) was stronger compared to clade 2 (0.40). Also, the mismatch distribution between individual and environmental coloration was more skewed within clade 1 compared to clade 2 (Fig. 4). Both results suggest tighter micro-habitat preferences within clade 1. It is also possible that clade 2 is competitively excluded by the more dominant clade 1 from the optimum micro-habitat, which may be responsible for the less skewed distribution for clade 2 (Fig. 4). Nonetheless, within both clades habitat-phenotype covariation was persistent. Thus, our results confirmed that in the admixture of the rocky (darker) and sandy (lighter) desert micro-habitats over North Africa, these sympatric clades may persist in ecological separation, with clade 1 being associated with brighter, sandy, areas and clade 2 with darker, rockier locations. Indeed, the phenotypic divergence strongly suggests that, to some extent, barriers to gene flow between the two putative species may have evolved, perhaps even as a result of ecologically-based differential selection. The role of geographic isolation in shaping this pattern is not obvious and cannot be completely excluded, but the persistence of both clades in sympatry, and the persistence of phenotype-environment association within both clades, suggests that geographic isolation might not be crucial for their coexistence [45]. Yet the distribution of the differences between mitochondrial haplotypes (Figure S1 in Appendix S1) and the observed overall genetic polymorphism (Table 1) suggest a slightly more recent demographic history of clade 2 versus clade 1, as already suggested in previous works [26] [31]. This might imply significant differences in the history of colonization of the North Africa from a previously locally confined population, but does not neglect micro-habitat specialization. Therefore, the ultimate test of sympatric versus parapatric divergence mechanisms awaits further investigation.

The novel remote sensing methodology presented here is applicable to other systems intended to investigate animal-habitat color covariation over large geographical scales. Using this technique, we have shown here the environmental basis of animal-habitat color matching as well as consistent phenotypic differences between sympatric genetic clades of African desert jerboas over a large geographical scale (the whole of North Africa). As dorsal fur coloration was significantly linked to the coloration of the environment within both clades, the process of natural selection might be persistent in contemporary populations of African jerboas. The sympatric distribution of the clades (regardless of possible allopatry in the past) suggests a specific micro-habitat preference, which likely has affected reproductive isolation, allowing the persistence of separate demes. This highlights the importance of an ecological mechanism, the evolution of camouflage, as a process affecting the evolution of distinct African desert jerboas. Tests on the extent of the reproductive isolation between these putative species will help to validate a speciation hypothesis. Further analyzes will also evaluate whether predation pressures are indeed differential between, and disruptive within, the clades, and whether or not it is based on ground color variation, which could cause an increase in the dorsal fur color polymorphism observed here.

## Supporting Information

Appendix S1 **Detailed information about data (samples GenBank affiliations, satellite images), remote sensing method and additional (partial Pearson correlations, mixed models, haplotypes mismatches) results included in the study.**
(PDF)

Appendix S2 **Representatives of genetic clades, upper individual belongs to clade 1 and lower to clade 2.**
(JPG)
