# Supplementary material for: Large Spatial Scale of the Phenotype-Environment Color Matching in Two Cryptic Species of African Desert Jerboas (Dipodidae: Jaculus)
Source: PLoS One. 2014 Apr 8;9(4):e94342. doi: 10.1371/journal.pone.0094342 (PMC3979769; doi:10.1371/journal.pone.0094342)
Supplement: Appendix S1 — Detailed information about data (samples GenBank affiliations, satellite images), remote sensing method and additional (partial Pearson correlations, mixed models, haplotypes mismatches) results included in the study. (PDF) [file pone.0094342.s001.pdf]

Table S1. Samples included in the study and their GenBank accession numbers. "Collection" - catalogue number of the collection where specimens are housed: D - CIBIO, Centro de Investigação em Biodiversidade e Recursos Genéticos, Universidade do Porto, USNM - Smithsonian Institution National Museum of Natural History, Washington D.C. and ZBSC - ZB collection. Est - colour (Col) values estimated.

Collection	Region	latitude	longitude	Col	cyt b GB	Collection	Region	latitude	longitude	Col	cyt b GB
D100	Mauritania	17.938317	-12.267117	-	JN214504	USNM325805	Libya	29.56667	24.70000	Est	KC663563
D1003	Mauritania	20.377967	-15.991150	-	JN214505	USNM325819	Libya	30.55000	18.46667	Est	KC663566
D101	Mauritania	17.938317	-12.267117	-	JN214506	USNM325821	Libya	31.18839	16.39905	Est	KC663569
D113	Mauritania	17.692550	-12.571133	-	JN214507	USNM325828	Libya	29.58852	24.86402	Est	KC663550
D117	Mauritania	17.392517	-13.452850	-	JN214508	USNM342028	Egypt	27.22097	30.80421	Est	KC663543
D1283	West. Sahara (Morocco)	22.446400	-16.448383	-	JN214509	USNM342030	Egypt	27.14105	31.38113	Est	KC663528
D144	West. Sahara (Morocco)	24.847250	-14.844483	-	JN214510	USNM342033	Egypt	28.31668	31.11665	Est	KC663540
D145	West. Sahara (Morocco)	25.245083	-14.821183	-	JN214511	USNM342034	Egypt	28.53958	30.56909	Est	KC663529
D1630	Mauritania	21.355050	-13.024950	-	JN214512	USNM342040	Egypt	29.69811	32.35410	Est	KC663521
D22	Mauritania	17.899250	-12.333783	-	JN214513	USNM342084	Sudan	15.22898	36.38786	Est	KC663515
D3055	Mauritania	17.895172	-11.716192	-	JN214514	USNM350066	Egypt	30.08107	31.58396	Est	KC663536
D3107	Mauritania	18.020882	-12.049943	-	JN214515	USNM350757	Iran	29.77051	50.56836	Est	KC663523
D320	Tunisia	33.014367	10.952350	-	JN214516	USNM401212	Mauritania	21.51667	-13.05000	Est	KC663571
D493	Mauritania	17.408217	-16.062283	-	JN214517	USNM475761	Morocco	31.90000	-4.48333	Est	KC663578
D506	Mauritania	19.438717	-14.753883	-	JN214518	USNM475764	Morocco	32.11667	-2.85000	Est	KC663573
D511	Mauritania	19.640667	-14.521700	-	JN214519	USNM475780	Morocco	31.95000	-3.55000	Est	KC663575
D52	Mauritania	17.225267	-7.068600	-	JN214520	USNM475783	Morocco	32.50000	-2.05000	Est	KC663574
D53	Mauritania	17.195050	-7.141233	-	JN214531	USNM475797	Morocco	31.83333	-4.58333	Est	KC663524
D535	West. Sahara (Morocco)	21.937267	-16.875067	-	JN214521	USNM475820	Morocco	32.15000	-1.25000	Est	KC663525
D536	West. Sahara (Morocco)	21.968500	-16.874133	-	JN214532	USNM475865	Morocco	30.30000	-5.93333	Est	KC663579
D541	West. Sahara (Morocco)	22.367417	-16.462367	-	JN214522	USNM475885	Morocco	32.68333	-3.08333	Est	KC663572
D549	West. Sahara (Morocco)	24.788033	-14.865367	-	JN214523	USNM482480	Algeria	32.45619	-0.57786	Est	KC663531
D576	Morocco	29.389283	-8.129267	-	JN214524	USNM482482	Algeria	30.05000	-2.21667	Est	KC663539
D577	Morocco	30.037683	-6.894050	-	JN214525	USNM482491	Algeria	26.86667	-0.96667	Est	KC663549
D578	Morocco	30.037683	-6.894050	-	JN214526	USNM482499	Algeria	23.56667	5.11667	Est	KC663557
D6	Morocco	28.468800	-11.021367	-	JN214527	USNM482502	Algeria	22.63333	5.73333	Est	KC663565
D684	Morocco	31.074917	-4.011250	-	JN214533	USNM482503	Algeria	22.93333	5.41667	Est	KC663577
D796	West. Sahara (Morocco)	24.686117	-14.861950	-	JN214528	USNM482504	Algeria	23.16667	5.11667	Est	KC663580
D800	West. Sahara (Morocco)	25.306200	-14.803383	-	JN214529	USNM482671	Niger	16.55000	6.86667	Est	KC663526
D945	Morocco	28.632800	-10.752650	-	JN214530	USNM482673	Niger	18.96667	5.96667	Est	KC663513
USNM279697	Kuwait	28.90207	48.08624	Est	KC663547	USNM482681	Niger	17.36667	6.71667	Est	KC663512
USNM282352	Kuwait	29.16563	46.91794	Est	KC663548	USNM482686	Niger	15.75000	6.60000	Est	KC663514
USNM282539	Egypt	30.08954	31.42529	Est	KC663535	USNM483105	Morocco	28.76667	-10.30000	-	KC663576
USNM283260	Egypt	30.31072	32.27830	Est	KC663538	ZBSC0013	Morocco	28.829343	-10.26583	-	JN214534
USNM297612	Sudan	19.53524	37.19247	Est	KC663517	ZBSC0019	Mauritania	20.996882	-16.28251	-	JN214535
USNM297613	Sudan	19.86667	37.18333	Est	KC663516	ZBSC0021	Mauritania	20.601737	-16.01246	-	JN214536
USNM317012	Egypt	22.52559	36.22540	Est	KC663530	ZBSC0027	Mauritania	16.56601	-14.19815	-	JN214537
USNM317013	Egypt	22.26930	36.39740	Est	KC663545	ZBSC0028	Mauritania	16.43478	-14.03688	-	JN214538
USNM317014	Egypt	22.29834	36.54394	Est	KC663532	ZBSC0064	Mauritania	18.900835	-15.41598	-	JN214539
USNM317015	Egypt	31.51679	25.60661	Est	KC663544	ZBSC0070	Mauritania	20.72443	-16.0571	Est	JN214540
USNM317017	Egypt	25.67288	32.77148	Est	KC663542	ZBSC0072	Mauritania	20.378803	-15.99129	-	JN214541
USNM317018	Egypt	30.40680	30.60440	Est	KC663533	ZBSC0081	West. Sahara (Morocco)	22.638767	-16.33688	Est	JN214542
USNM317020	Egypt	30.49886	30.79409	Est	KC663537	ZBSC0082	West. Sahara (Morocco)	24.296773	-15.33347	-	JN214543
USNM317028	Egypt	25.25778	32.45711	Est	KC663541	ZBSC0083	West. Sahara (Morocco)	24.629537	-14.94513	-	JN214544
USNM317041	Egypt	30.21899	30.89907	Est	KC663520	ZBSC0084	West. Sahara (Morocco)	25.321792	-14.79502	-	JN214545
USNM317047	Egypt	29.49410	30.40152	Est	KC663518	ZBSC196	West. Sahara (Morocco)	24.00569	-15.61102	-	KC663499
USNM317049	Egypt	30.63450	29.83903	Est	KC663519	ZBSC197	West. Sahara (Morocco)	22.82889	-16.24960	-	KC663500
USNM317050	Egypt	30.09904	31.58075	Est	KC663534	ZBSC198	West. Sahara (Morocco)	22.55694	-16.37038	-	KC663501
USNM317059	Egypt	30.40680	30.60440	Est	KC663522	ZBSC218	Mauritania	21.43810	-12.98000	Est	KC663502
USNM317065	Egypt	24.00977	32.82735	Est	KC663527	ZBSC219	Mauritania	21.35222	-13.03868	Est	KC663503
USNM317068	Egypt	28.56239	33.96295	Est	KC663546	ZBSC224	Mauritania	20.55665	-12.57188	Est	KC663582
USNM319773	Libya	24.18333	23.31667	Est	KC663552	ZBSC226	Mauritania	20.50774	-12.83122	Est	KC663504
USNM321863	Libya	32.06458	11.35046	Est	KC663567	ZBSC240	Mauritania	20.25413	-13.29555	Est	KC663505
USNM321864	Libya	30.75000	11.51667	Est	KC663551	ZBSC241	Mauritania	20.25278	-13.31065	-	KC663506
USNM322762	Libya	29.08894	15.89575	Est	KC663564	ZBSC242	Mauritania	20.01584	-13.88718	Est	KC663583
USNM322767	Libya	27.22384	14.66284	Est	KC663559	ZBSC243	Mauritania	19.65052	-14.50437	-	KC663507
USNM322770	Libya	26.76667	14.00000	Est	KC663555	ZBSC244	Mauritania	19.65052	-14.50437	-	KC663508
USNM322788	Libya	24.95334	10.20575	Est	KC663556	ZBSC245	Mauritania	19.65052	-14.50437	-	KC663509
USNM322798	Libya	25.90257	13.89004	Est	KC663558	ZBSC256	Mauritania	17.59071	-12.84784	Est	KC663581
USNM322803	Libya	27.00000	14.45000	Est	KC663568	ZBSC265	Mauritania	18.09402	-12.13151	-	KC663510
USNM322809	Libya	27.55000	14.25000	Est	KC663553	ZBSC267	West. Sahara (Morocco)	22.13568	-16.56978	-	KC663511
USNM322811	Libya	27.53333	13.20000	Est	KC663554	Allactaga elater		-	-	-	AJ389534
USNM325770	Libya	32.41667	13.05000	Est	KC663560	complete mtDNA		-	-	-	NC005314
USNM325774	Libya	29.25000	21.23333	Est	KC663561	Dipus sagitta		-	-	-	AM407909
USNM325789	Libya	25.75000	21.15000	Est	KC663570	Jaculus orientalis		-	-	-	JN652663
USNM325802	Libya	29.75000	24.55000	Est	KC663562						

Table S2. Spatial (Path and Row) and temporal (Date) information of Landsat (Sat: L7 – Landsat 7 ETM$^+$, L5 - Landsat 5 TM) scenes used in this study.

Country	Path	Row	Date	Sat	Date	Sat	Country	Path	Row	Date	Sat	Date	Sat
Pakistan	155	41	23.4.2003	L7	6.9.2000	L5	Tunisia	190	37	9.7.2003	L5	20.6.2002	L5
Iran	157	41	21.4.2003	L7	25.7.2000	L5	Niger	190	48	11.3.2003	L7	13.11.2000	L7
	158	36	28.4.2003	L7	30.6.2000	L5		190	50	14.5.2003	L7	21.5.2000	L7
	160	39	26.4.2003	L7	28.6.2000	L5		191	47	5.5.2003	L7	16.8.2000	L7
	164	36	5.3.2003	L7	1.2.2000	L5	Algeria	191	45	16.7.2003	L5	27.6.2002	L5
	164	40	24.5.2003	L7	19.8.2000	L7		192	44	7.7.2003	L5	17.5.2002	L5
Kuwait	165	40	29.4.2003	L7	17.7.2000	L5		194	42	21.7.2003	L5	31.7.2001	L5
	166	40	22.5.2003	L7	22.6.2000	L5		195	41	12.7.2003	L5	22.5.2002	L5
Sudan	171	46	9.5.2003	L7	8.5.2000	L5		195	42	12.7.2003	L5	7.6.2002	L5
	171	47	9.5.2003	L7	8.5.2000	L5		197	38	26.7.2003	L5	5.6.2002	L5
	171	49	9.5.2003	L7	21.3.2000	L5		198	39	1.7.2003	L5	14.6.2000	L7
	175	45	5.5.2003	L7	5.6.2000	L5	Morocco	198	37	1.7.2003	L5	28.6.2002	L5
Egypt	172	45	11.7.2003	L5	18.7.2000	L5		198	38	1.7.2003	L5	28.6.2002	L5
	173	43	18.7.2003	L5	25.7.2000	L5		199	36	24.7.2003	L5	19.6.2002	L5
	174	40	9.7.2003	L5	14.6.2000	L5		199	37	24.7.2003	L5	19.6.2002	L5
	175	40	16.7.2003	L5	5.6.2000	L5		199	38	25.8.2003	L5	19.6.2002	L5
	175	42	16.7.2003	L5	5.6.2000	L5		199	39	24.7.2003	L5	25.5.2002	L5
	176	39	7.7.2003	L5	3.7.2000	L5		200	37	31.7.2003	L5	25.5.2002	L5
	176	40	7.7.2003	L5	3.7.2000	L5		200	38	31.7.2003	L5	25.7.2001	L5
	176	41	23.7.2003	L5	3.7.2000	L5		200	39	31.7.2003	L5	17.6.2002	L5
	176	43	7.7.2003	L5	3.7.2000	L5		201	39	6.7.2003	L5	17.6.2002	L5
	177	38	14.7.2003	L5	30.4.1999	L5		201	40	6.7.2003	L5	8.6.2002	L5
	177	39	14.7.2003	L5	30.4.1999	L5		202	40	27.7.2003	L5	5.6.2000	L7
	177	40	30.7.2003	L5	30.4.1999	L5		202	41	16.4.2003	L7	23.4.2000	L7
	178	38	21.7.2003	L5	5.4.1999	L5		203	40	20.7.2003	L5	10.12.2000	L7
	178	39	21.7.2003	L5	5.4.1999	L5		205	43	21.4.2003	L7	15.6.2000	L7
	179	38	12.7.2003	L5	23.6.2002	L5		205	45	5.4.2003	L7	15.6.2000	L7
	180	38	19.7.2003	L5	13.5.2002	L5	Mauritania	199	48	26.3.2003	L7	24.8.2000	L7
Lybia	180	43	3.7.2003	L5	13.5.2002	L5		202	48	31.3.2003	L7	30.9.2000	L7
	181	39	26.7.2003	L5	17.7.2000	L5		203	41	7.4.2003	L7	7.10.2000	L7
	182	37	17.7.2003	L5	27.7.2001	L5		203	45	22.3.2003	L7	1.6.2000	L7
	182	38	1.7.2003	L5	27.7.2001	L5		203	46	23.4.2003	L7	5.9.2000	L7
	182	40	17.7.2003	L5	27.7.2001	L5		203	48	6.3.2003	L7	5.9.2000	L7
	182	42	17.7.2003	L5	27.7.2001	L5		203	49	23.4.2003	L7	5.9.2000	L7
	183	39	8.7.2003	L5	25.5.2002	L5		204	45	14.4.2003	L7	24.6.2000	L7
	184	38	15.7.2003	L5	25.5.2002	L5		204	46	14.4.2003	L7	24.6.2000	L7
	184	39	15.7.2003	L5	17.5.2002	L5		204	47	14.4.2003	L7	24.6.2000	L7
	185	39	6.7.2003	L5	23.5.2002	L5		204	48	13.3.2003	L7	15.11.2000	L7
	186	38	13.7.2003	L5	23.5.2002	L5		205	46	5.4.2003	L7	30.5.2000	L7
	186	40	29.7.2003	L5	28.8.2000	L5		205	47	5.4.2003	L7	30.5.2000	L7
	187	38	4.7.2003	L5	28.8.2000	L5		205	48	5.4.2003	L7	15.6.2000	L7
	187	41	4.7.2003	L5	28.8.2000	L5		206	45	28.4.2003	L7	6.6.2000	L7
	187	42	4.7.2003	L5	21.5.2002	L5		206	46	28.4.2003	L7	9.8.2000	L7
	188	38	27.7.2003	L5	29.6.2002	L5							
	188	42	27.7.2003	L5	29.6.2002	L5							
	189	38	2.7.2003	L5	20.5.2000	L7							
	189	43	18.7.2003	L5	24.6.2000	L7							

Surface reflectance calculation from satellite images

1.1 Remote Sensing data

Satellite images from the Landsat 5 Thematic Mapper (TM) and Landsat 7 Enhanced Thematic Mapper (ETM^+) series were obtained through the Global Visualization Viewer (GLOVIS; http://glovis.usgs.gov/) of the United States Geological Survey (USGS). The scenes were selected according to the samples location. The time period corresponded to the dry seasons of each region from 2003, and only images with a cloud cover lower than 10% were chosen. The final dataset comprised 180 satellite images (Table S2), with a spatial resolution of 30 m. The Landsat images were georeferenced by the USGS (http://landsat.usgs.gov/). The analyses were performed for the bands 1, 2 and 3 (blue, green and red, respectively) of every Landsat image. All analyses were developed in the ArcGIS 9.3 software (ESRI, 2008) on the WGS 1984 UTM datum.

1.2 Atmospheric correction

An absolute atmospheric correction was performed in order to eliminate atmospheric effects and for obtaining the real surface reflectances. The absolute atmospheric correction was conducted in two steps: 1) the conversion of 8-bit satellite-quantised calibrated digital numbers (DN) to at-satellite radiance; and 2) the conversion of at-satellite radiance to atmospherically-corrected surface reflectance.

1.2.1 Conversion of DN to at-satellite radiance

The Landsat 5 TM and Landsat 7 ETM+ images were converted to at-satellite radiance through the application of Eq.(1),

$$L_\lambda = G_{rescale} \times Q_{cal} + B_{rescale} \qquad (1)$$

where

L_λ = Spectral radiance at the sensor's aperture [W/(m^2 sr μm)]

$G_{rescale}$ = Band-specific rescaling gain factor [(W/(m^2 sr μm))/DN]

Q_{cal} = Quantised calibrated pixel value [DN]

$B_{rescale}$ = Band-specific rescaling bias factor [W/(m^2 sr μm)]

The $G_{rescale}$ and $B_{rescale}$ parameters published by Chander *et al*. (2009), were used for each specific band. The calculations were conducted through the Spatial Analyst extension for ArcGIS.

1.2.2 Conversion of at-satellite radiance to surface reflectance

The at-satellite radiances were converted to surface reflectances (assuming a uniform Lambertian surface under cloudless conditions) using Eq.(2),

$$\rho = \pi (L_\lambda - L_p) / T_v [E_0 \times \cos(\theta) \times T_z + E_{down}] \quad (2)$$

where

ρ = Estimated surface reflectance

L_λ = Spectral radiance at the sensor's aperture [W/(m^2 sr μm)]

L_p = Path radiance [W/(m^2 sr μm)]

T_v = Atmospheric transmittance from the target toward the sensor

E_0 = Exoatmospheric solar constant [W/(m^2 sr μm)] (corrected for solar distance)

θ = Solar zenith angle [degrees]

T_z = Atmospheric transmittance in the illumination direction

E_{down} = Downwelling diffuse irradiance [W/(m^2 μm)]

The cosine of the solar zenith angle is equal to the sine of the solar elevation angle, which is a parameter that is stored in the Level 1 product header file (.MTL) of each Landsat scene (Chander *et al*., 2009). The E_0 resulted from the correction of the exoatmospheric solar constant for the Earth-Sun distance. Both parameters were defined according to the values published by Chander *et al*. (2009). The parameters T_v, T_z and E_{down} were obtained through the first Dark Object Subtraction (DOS) approach proposed by Song *et al*., (2001). The DOS approach has been referenced as a simple and accurate method in the absolute atmospheric correction of satellite images (Song *et al*., 2001; Schroeder *et al*., 2006). The L_p was estimated using Eq.(3),

$$L_p = G_{rescale} \times DN_{dark} + B_{rescale} - 0.01\ [E_0 \times \cos(\theta) \times T_z + E_{down}]\ T_v / \pi \qquad (3)$$

where

DN_{dark} = Darkest DN value in each spectral band with at least one thousand pixels (Teillet and Fedosejevs, 1995; McDonald *et al*., 1998)

All the calculations for the three specific bands of each Landsat scene were performed by the Spatial Analyst.

1.3 Reflectances of the Jaculus *samples*

Buffers with 1, 5 and 10 km radius were built around each *Jaculus* sample, using the Proximity tool of the Analysis extension for ArcGIS. For obtaining the surface background reflectance of the samples, the atmospherically corrected images were intersected with the buffers. The minimum, maximum, mean and standard deviation of the reflectance values comprised by the buffers were calculated and extracted through the Zonal Statistics tool of Spatial Analyst.

References

Chander, G., Markham, B.L., Helder, D.L., 2009. Summary of current radiometric calibration coefficients for Landsat MSS, TM, ETM^+, and EO-1 ALI sensors. *Remote Sensing of Environment*, *113*, 893–903.

ESRI, 2008. *Arcmap 9.3*. Environmental Systems Research Institute, Inc., Redlands, California.

McDonald, A.J., Gemmell, F.M., Lewis, P.E., 1998. Investigation of the utility of spectral vegetation indices for determining information on coniferous forests. *Remote Sensing of Environment*, 66, 250–272.

Schroeder, T.A., Cohen, W.B., Song, C., Canty, M.J., Yang, Z., 2006. Radiometric correction of multi-temporal Landsat data for characterization of early successional forest patterns in western Oregon. *Remote Sensing of Environment*, *103*, 16–26.

Song, C., Woodcock, C. E., Seto, K. C., Pax-Lenney, M., Macomber, S. A., 2001. Classification and change detection using Landsat TM data: When and how to correct atmospheric effects. *Remote Sensing of Environment*, *75*, 230−244.

Teillet, P.M., Fedosejevs, G., 1995. On the dark target approach to atmospheric correction of remotely sensed data. *Canadian Journal of Remote Sensing*, *21*, 373–387.

Table S3. Partial Pearson correlations between individual and environmental colours, accounting for sample affiliation either to museum or to field collections.

	colour	r	t	df	p
All data					
	red	0.39	3.65	77	0.0002
	green	0.30	2.76	77	0.0057
	blue	0.23	2.08	77	0.037
Clade 1					
	red	0.44	3.21	47	0.0013
	green	0.31	2.18	47	0.029
	blue	0.33	2.31	47	0.021
Clade 2					
	red	0.39	2.21	30	0.027
	green	0.41	2.32	30	0.020
	blue	-0.16	-0.82	30	0.41

Table S4. Model components and parameters from AIC model selection procedure. Fur luminosity and RGB colours, the dependent variables, were analysed in separate mixed models that included clade (two clades) and museum/field affiliations as fixed factors, environmental luminosity and RGB colours, and their factorial interactions, as continuous predictors, and random factor of individual code. Best models depicted by AIC are in bold.

Museum samples only	Models parameters				
Dependent and independent variables	df	logLik	AICc	Delta	Weight
Fur Luminosity					
**Clade+Red+Green**	**6**	**-257.6**	**556.4**	**0.00**	**0.31**
Clade+Red+Blue+Luminosity	7	-252.6	557.6	1.20	0.17
Clade+Red+Blue+Green	7	-253.1	557.6	1.20	0.17
Clade+Red+Green+Luminosity	7	-250.9	557.6	1.20	0.17
Clade+Blue+Green+Luminosity	7	-251.9	557.6	1.20	0.17
Fur Red					
**Clade+Red+Green**	**6**	**-264.6**	**571.2**	**0.00**	**0.39**
Clade+Red+Blue+Luminosity	7	-259.8	573.0	1.84	0.15
Clade+Red+Blue+Green	7	-260.3	573.0	1.84	0.15
Clade+Red+Green+Luminosity	7	-258.1	573.0	1.84	0.15
Clade+Blue+Green+Luminosity	7	-259.1	573.0	1.84	0.15
Fur Blue					
**Clade+Red+Green**	**6**	**-244.5**	**528.4**	**0.00**	**0.23**
Clade+Red+Luminosity	6	-244.1	528.6	0.22	0.2
Clade+Red+Blue+Luminosity+C.*B.	8	-233.6	528.7	0.34	0.19
Clade+Red+Blue+Green+C.*B.	8	-234.1	528.7	0.34	0.19
Clade+Blue+Green+Luminosity+C.*B.	8	-232.9	528.7	0.34	0.19
Fur Green					
**Clade+Red+Green**	**6**	**-257.5**	**556.1**	**0.00**	**0.27**
Clade+Red+Luminosity	6	-257.3	556.7	0.65	0.2
Clade+Red+Blue	6	-260.0	556.9	0.76	0.19
Clade+Red+Blue+Luminosity	7	-252.3	557.0	0.88	0.17
Clade+Red+Blue+Green	7	-252.9	557.0	0.89	0.17
Museum and African samples only					
Fur Luminosity					
**Clade+Red+Green**	**6**	**-245.8**	**533.4**	**0.00**	**0.25**
Clade+Red	5	-253.2	533.5	0.09	0.24
Clade+Luminosity	5	-252.9	534.1	0.65	0.18
Clade+Red+Blue	6	-248.4	534.2	0.76	0.17
Clade+Red+Blue+Blue*Clade	7	-242.6	534.5	1.11	0.15
Fur Red					
**Clade+Red+Green**	**6**	**-252.7**	**548.2**	**0.00**	**0.33**
Clade+Red	5	-260.5	549.0	0.81	0.22
Clade+Red+Green+C.*G.+C.*R.	8	-238.3	549.2	1.02	0.2
Clade+Red+Blue+Luminosity	7	-247.7	550.0	1.84	0.13
Clade+Red+Blue+Green	7	-248.3	550.0	1.84	0.13
Fur Blue					
**Clade+Red+Blue+C.*B.**	**7**	**-229.1**	**505.4**	**0.00**	**0.29**
Clade+Red	5	-240.1	506.2	0.80	0.19
Clade+Luminosity	5	-239.6	506.2	0.81	0.19
Clade+Red+Blue	6	-235.4	506.5	1.05	0.17
Clade+Red+Blue+Luminosity+C.*B.	8	-222.0	506.6	1.19	0.16
Fur Green					
**Clade+Red**	**5**	**-252.7**	**532.6**	**0.00**	**0.23**
Clade+Luminosity	5	-252.3	532.8	0.19	0.21
Clade+Red+Green	6	-245.5	532.9	0.29	0.2
Clade+Red+Blue	6	-247.8	533.0	0.38	0.19
Clade+Red+Blue+C.*B.	7	-241.9	533.1	0.43	0.18

Table S5. Relative variable importance in explaining variation in animals' overall dorsal fur luminosity and RGB colours. Fur luminosity and RGB colours, the dependent variables, were analysed in separate mixed models that included clade (two clades) and museum/field affiliations as fixed factors, environmental luminosity and RGB colours, and their factorial interactions, as continuous predictors, and random factor of individual code. Average values in bold are above 0.6.

All data	Clade	M/F	Green	Red	Lum.	Blue		M/F*R.	C.*R.	M/F*G.	C.*G.	C.*L.
Fur Luminosity	1	1	0.8	0.8	0.5	0.5		0	0	0	0	0
Fur Red	1	1	1	1	0	0		0.5	0.3	0.2	0.1	0
Fur Blue	1	0	0.7	0.3	0.8	0.3		0	0	0.0	0.2	0.4
Fur Green	1	1	0.6	0.8	0.4	0.6		0	0	0	0	0
average	**1.00**	**0.75**	**0.78**	**0.73**	0.43	0.35		0.13	0.07	0.05	0.09	0.10
Museum sample	Clade		Green	Red	Lum.	Blue	C.*B.					
Fur Luminosity	1		0.83	0.83	0.52	0.52	0					
Fur Red	1		0.85	0.85	0.46	0.46	0					
Fur Blue	1		0.81	0.61	0.58	0.57	0.57					
Fur Green	1		0.64	0.8	0.37	0.53	0					
average	**1.00**		**0.78**	**0.77**	0.48	0.52	0.14					
Museum, African samples	Clade		Green	Red	Lum.	Blue	C.*B.		C.*R.		C.*G.	
Fur Luminosity	1		0.25	0.82	0.18	0.32	0.15		0		0	
Fur Red	1		0.65	0	0	0.26	0.13		0.2		0.2	
Fur Blue	1		0	0.81	0.35	0.62	0.45		0		0	
Fur Green	1		0.2	0.79	0.21	0.37	0.18		0		0	
average	**1.00**		0.28	**0.61**	0.19	0.39	0.23		0.05		0.05	

Table S6. Best model results of mixed models analyses for variations in animals' overall dorsal fur luminosity and RGB colours depicted from AIC model selection procedure.

Dependent	Independent variables	Value	SE	DF	t-value	p-value
Museum samples only						
Fur Luminosity						
	(Intercept)	138.3	11.0	64	12.54	<.0001
	Clade	-11.5	3.5	64	-3.28	0.0017
	Green10M	-342.9	167.1	64	-2.05	0.044
	Red10M	254.9	78.3	64	3.26	0.0018
Fur Red						
	(Intercept)	184.0	12.3	64	14.96	<.0001
	Clade	-15.3	3.9	64	-3.91	0.0002
	Green10M	-412.1	186.42	64	-2.21	0.031
	Red10M	290.1	87.3	64	3.32	0.0015
Fur Blue						
	(Intercept)	92.4	9.0	64	10.29	<.0001
	Clade	-6.7	2.9	64	-2.34	0.022
	Green10M	-264.6	136.1	64	-1.94	0.056
	W10km	200.8	63.7	64	3.15	0.0025
Fur Green						
	(Intercept)	124.9	11.0	64	11.35	<.0001
	Clade	-10.5	3.5	64	-3.00	0.0001
	Green10M	-323.0	166.8	64	-1.94	0.057
	Red10M	247.6	78.1	64	3.17	0.0023
Museum and African samples only						
Fur Luminosity						
	(Intercept)	124.0	12.5	62	9.93	<.0001
	Clade	-10.8	3.8	62	-2.84	0.01
	Red10M	127.3	34.0	62	3.75	0.0004
Fur Red						
	(Intercept)	168.0	14.1	62	11.95	<.0001
	Clade	-14.7	4.3	62	-3.43	0.0011
	Red10M	133.6	38.3	62	3.49	00009
Fur Blue						
	(Intercept)	80.6	10.1	62	7.96	<.0001
	Clade	-56.0	3.1	62	-1.93	0.0579
	Red10M	104.4	27.5	62	3.79	0.0003
	Clade:Blue10M					
Fur Green						
	(Intercept)	111.0	12.4	62	8.95	<.0001
	Clade	-9.8	3.8	62	-2.58	0.0122
	Red10M	128.6	33.7	62	3.81	0.0003

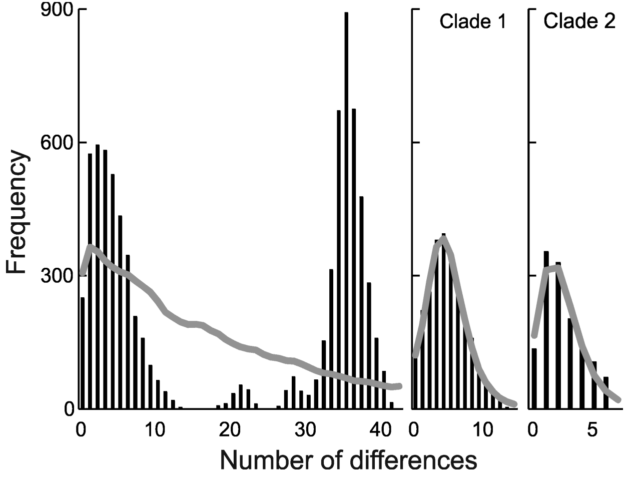

Observed (bars) and expected (lines) differences between cytochrome b haplotypes.
