# Supplementary figures and images for: Large Spatial Scale of the Phenotype-Environment Color Matching in Two Cryptic Species of African Desert Jerboas (Dipodidae: Jaculus)
Source: PLoS One. 2014 Apr 8;9(4):e94342. doi: 10.1371/journal.pone.0094342 (PMC3979769; doi:10.1371/journal.pone.0094342)

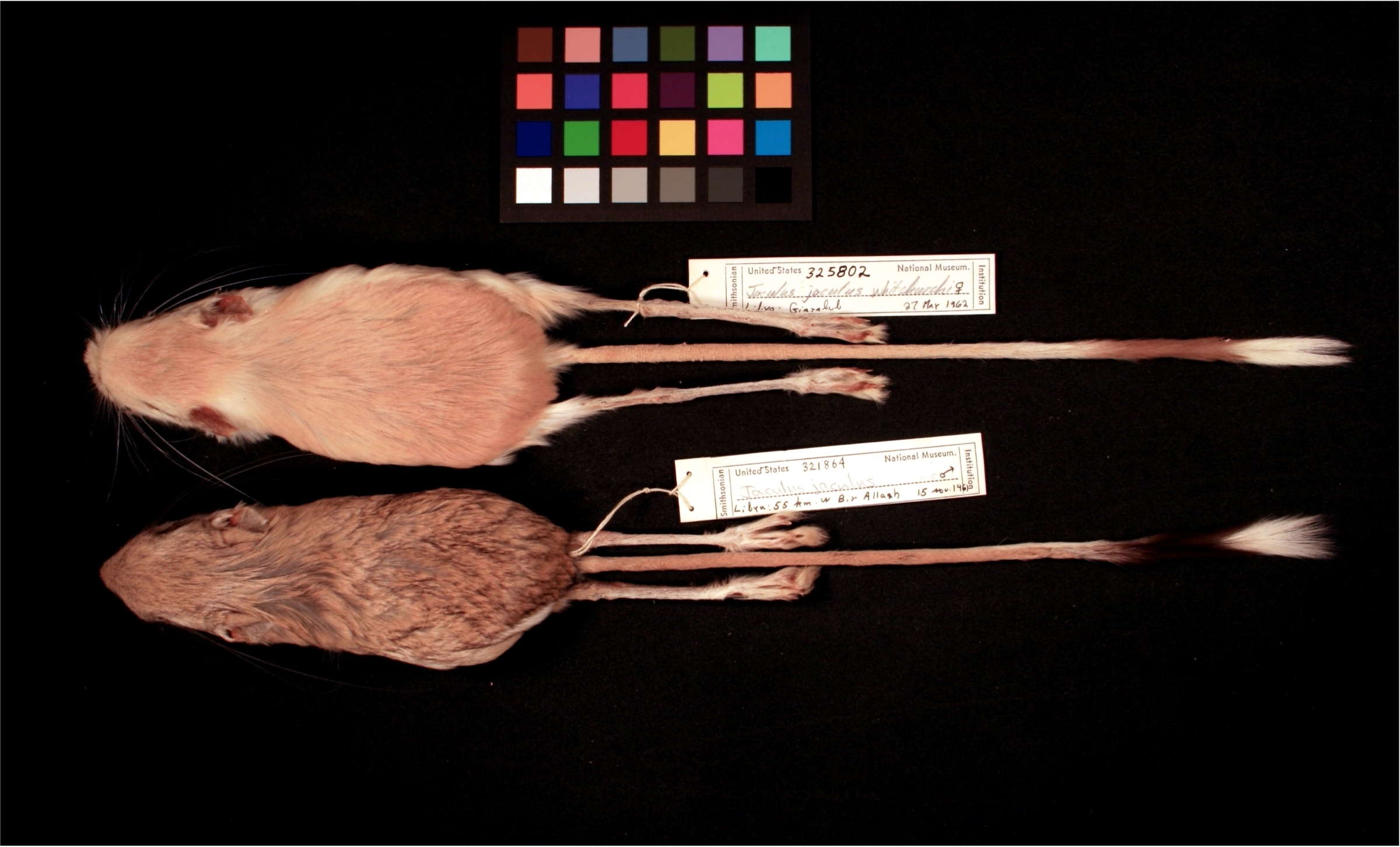

Supplement: Appendix S2 — Representatives of genetic clades, upper individual belongs to clade 1 and lower to clade 2. (JPG) [file pone.0094342.s002.jpg]
